# Choanal Narrowing in Children With Down Syndrome: A Retrospective Case Series

**DOI:** 10.7759/cureus.93223

**Published:** 2025-09-25

**Authors:** Ahmed H Ali, German P Digoy

**Affiliations:** 1 Otolaryngology, University of Oklahoma Health Sciences Center, Oklahoma City, USA; 2 Pediatric Otolaryngology, Oklahoma State University Center for Health Sciences, Tulsa, USA

**Keywords:** choanal narrowing, choanal stenosis, down syndrome, pediatric airway obstruction, trisomy 21

## Abstract

A retrospective case series was conducted on pediatric patients with Down syndrome who underwent choanal narrowing repair at a tertiary pediatric center between May 2017 and July 2024. Of the 84 patients who underwent repair, 13 children with Down syndrome (mean age: 2.54 years) were identified, representing 15.5% of the cohort. All had choanal narrowing (30%-80% stenosis, mean: 62%) and underwent concurrent airway procedures including adenoidectomy (n = 9), turbinate reduction (n = 7), and adenotonsillectomy (n = 3). Caregivers reported improvement in nasal breathing and obstructive sleep apnea (OSA) symptoms in all patients and improvement in snoring in 12 of 13 (92%) patients. One patient (8%) required revision surgery for restenosis. Children with Down syndrome represented a substantial proportion (15.5%) of those undergoing choanal narrowing repair, compared to ~0.2% prevalence in the general population. These findings suggest choanal narrowing may be more common in Down syndrome than previously recognized and should be considered when evaluating airway obstruction in this population. Prospective studies using objective outcome measures are needed.

## Introduction

Down syndrome (DS), caused by trisomy of chromosome 21, is one of the most common genetic disorders in children, affecting approximately one in 582 live births [1]. Children with DS face a variety of medical challenges including cardiac anomalies [2], gastrointestinal anomalies [3], and a significant predisposition to obstructive sleep apnea (OSA). OSA affects between 55% and 97% of children with DS, depending on the presence of snoring [4,5].

Multiple anatomical factors contribute to airway obstruction in DS, including midfacial hypoplasia, macroglossia, and small nasopharyngeal dimensions [6]. Choanal narrowing may represent an underrecognized contributor to airway obstruction in this population, yet it has received limited attention in the literature compared to other airway contributors such as adenotonsillar hypertrophy. The objective of this study was to describe the clinical presentation, intraoperative characterization, concurrent airway procedures, and caregiver-reported outcomes after endoscopic repair of choanal narrowing in children with DS treated at a single tertiary pediatric center (May 2017-July 2024).

## Materials and methods

Study design and patient selection

This IRB-approved, Health Insurance Portability and Accountability Act (HIPAA)-waived retrospective consecutive case series was conducted at a single tertiary pediatric center in Oklahoma City, Oklahoma. We included children with DS, confirmed by clinician documentation, who underwent endoscopic choanal narrowing repair from May 2017 to July 2024, had ≥30% stenosis on endoscopic visual estimate by the attending surgeon, and had ≥6 months postoperative follow-up. Eligible patients were identified through electronic medical records and operative logs. Charts were manually reviewed to verify eligibility and outcomes. No patients were excluded or lost to follow-up. The center is a regional referral site for complex pediatric airway disorders; 8/13 (62%) of patients were referred by external otolaryngologists.

Definition and assessment of choanal narrowing

Choanal narrowing was defined as a ≥30% reduction in posterior choanal cross-sectional diameter relative to age-appropriate norms. The degree of narrowing was estimated intraoperatively by comparative visual assessment using a calibrated 2.7-mm endoscope and standard suction catheter, performed by a single attending pediatric otolaryngologist (with 25 years of experience). This narrowing was further defined as an obstruction causing symptoms that significantly affected the patient’s quality of life. All patients had preoperative nasal endoscopy confirming bilateral choanal narrowing.

Data collection

Demographic data, degree of choanal narrowing, preoperative sleep study results (when available), and concurrent surgical procedures were recorded. All patients had previously trialed age- and symptom severity-based medical management, including intranasal corticosteroids in appropriate cases. Surgery was pursued due to persistent or progressive symptoms despite medical therapy. The decision to perform multilevel airway surgery, including adenoidectomy, turbinate reduction, and/or tonsillectomy, was individualized for each patient based on comprehensive clinical evaluation, airway endoscopy, and intraoperative findings. While preoperative sleep studies (when available) contributed to clinical assessment, the extent of surgical intervention was determined by the overall pattern of airway obstruction commonly seen in children with DS, rather than solely by OSA severity. Data were analyzed descriptively. Continuous variables are reported as mean ± standard deviation (SD) and range. Categorical variables are reported as counts and percentages. No inferential statistics were performed given the small sample size and descriptive nature of this study.

Outcome assessment

The primary outcome was caregiver-reported symptom improvement in nasal obstruction, snoring, and OSA symptoms at routine follow-up visits at approximately six months postoperatively. Improvement was documented based on the physician's interview and noted in the clinic documentation. No validated sleep questionnaires were used, and postoperative polysomnography was not routinely performed.

Surgical technique

All procedures were performed under general anesthesia with endoscopic visualization. A 0° endoscope was used for visualization. Stenotic tissue (fibrous and bony in all patients) was excised using cold steel instrumentation and a microdebrider. In cases of bony narrowing, limited resection of the posterior vomer and medial pterygoid plates was performed using small up and backbiting forceps. Care was taken to preserve the mucosa when feasible. Hemostasis was achieved using bipolar cautery and monopolar suction cautery. Bilateral narrowing was addressed in all patients. No stents were used postoperatively.

## Results

Of the 84 patients who underwent choanal narrowing repair during the study period, 13 (15.5%) had DS. This subgroup included nine males (69%) and four females (31%), with ages ranging from four months to six years (mean: 2.54 ± 2.25 years). The degree of choanal narrowing ranged from 30% to 80%, with a mean narrowing of 62% ± 15%, estimated visually by the attending surgeon intraoperatively. The majority of cases were regional referrals, consistent with our institution’s role as a tertiary airway center. Of note, eight of the 13 patients (62%) were referred from outside otolaryngologists.

All patients underwent concurrent airway procedures during the same anesthetic event, reflecting the complex, multilevel airway management often required in this population. The most common additional procedures included adenoidectomy in nine patients (69%) and turbinate reduction in seven patients (54%). Adenotonsillectomy was performed in three patients (23%), supraglottoplasty in two patients (15%), and lingual tonsil coblation in one patient (8%). Bilateral myringotomy with tube placement and other minor airway interventions were performed in multiple patients. Notably, none of the patients in this cohort underwent isolated choanal narrowing repair without additional procedures, highlighting the multifactorial nature of airway obstruction in children with DS.

**Figure 1:**
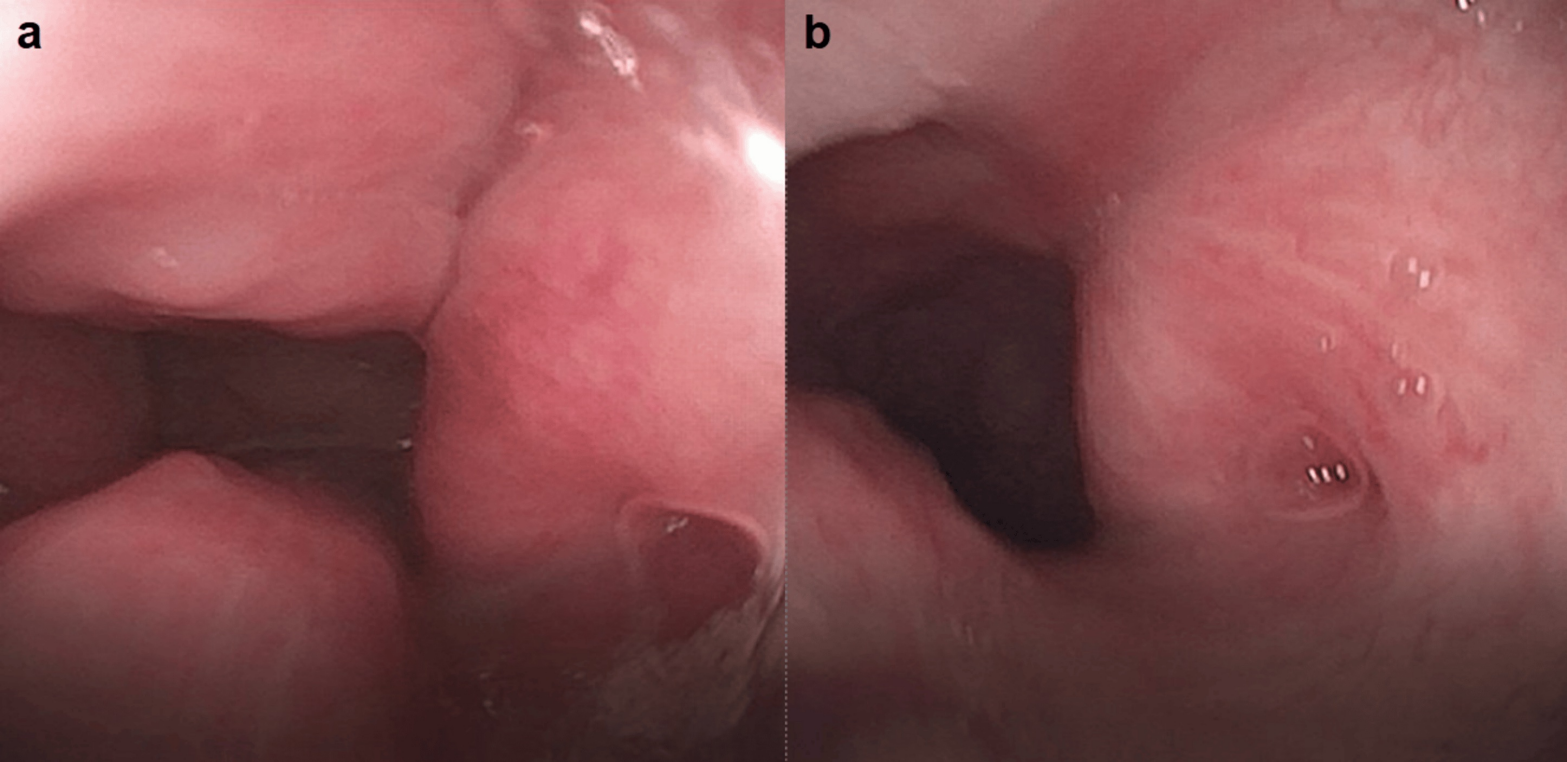
(a) Preoperative nasal endoscopy of a 28-month-old male with Down syndrome showing choanal narrowing. (b) Six-month postoperative nasal endoscopy of the same patient demonstrating improved choanal patency following surgical repair.

Caregiver-reported outcomes demonstrated consistent postoperative improvement across multiple domains at the time of follow-up. All 13 patients (100%) had improvement in nasal breathing and OSA symptoms, while 12 of 13 patients (92%) reported improvement in snoring. One patient required revision choanal repair 15 months after the initial surgery due to restenosis, representing a revision rate of 8%. Figure 1 illustrates the pre- and postoperative nasal endoscopy findings in a representative patient. Table 1 summarizes the demographic data, available preoperative sleep study results, concurrent procedures, and postoperative outcomes for all 13 patients.

**Table 1:** Clinical characteristics, concurrent procedures, and postoperative outcomes in Down syndrome patients undergoing choanal narrowing repair Continuous variables are presented as mean ± SD (range). Categorical variables are presented as N (%). Outcome measures are based on caregiver reports at postoperative follow-up visits. AHI: Apnea-hypopnea index; REM: Rapid eye movement; OSA: Obstructive sleep apnea.

Patient	Gender	Age at Surgery	Sleep Study Pre-surgery AHI (Overall/REM)	Post-op Snoring	Post-op OSA Symptoms	Post-op Nasal Breathing	Concurrent Procedures
1	F	4 months	18.6/28.3	Improved	Improved	Improved	Upper lip frenulum release and adenoidectomy
2	M	12 months	12.7/43.8	Improved	Improved	Improved	Laryngeal cleft closure, turbinate reduction, adenotonsillectomy, and supraglottoplasty
3	M	5 years	4.7/7.2	Improved	Improved	Improved	Turbinate reduction, adenoidectomy, and vocal cord Botox
4	F	3 years	4.6/24	Improved	Improved	Improved	Turbinate reduction and adenoidectomy
5	M	8 months	No sleep study	Improved	Improved	Improved	Supraglottoplasty and adenoidectomy
6	F	2 years	2.8/4.74	Improved	Improved	Improved	Laryngoplasty, septoplasty, turbinate reduction, and adenoidectomy
7	M	11 months	2/6.4	Improved	Improved	Improved	Bilateral tube insertion, laryngoplasty, and adenoidectomy
8	M	12 months	41/70	N/A	Improved	Improved	Upper lip frenulum release, adenotonsillectomy, and bilateral tubes
9	M	4 months	No sleep study	Improved	Improved	Improved	Tongue-to-lip adhesion release, bilateral tubes, and adenoidectomy
10	M	5 months	No sleep study	Improved	Improved	Improved	Adenoidectomy
11	M	6 years	10.9/19.2	Improved	Improved	Improved	Lingual tonsil coblation, nasal turbinate reduction, adenoidectomy, and bilateral tubes
12	F	6 years	2.9/14.9	Improved	Improved	Improved	Adenotonsillectomy, bilateral tubes, and turbinate reduction
13	M	4 years	12.3/30.7	Improved	Improved	Improved	Tonsillectomy

## Discussion

This study describes a single-center experience managing choanal narrowing in children with DS. At our institution, 13 of the 84 patients (15.5%) who underwent surgical repair for choanal narrowing had DS, whereas DS affects roughly 0.2% of the general pediatric population [1]. This represents a ~77-fold greater proportion of DS among our surgical patients than would be expected based on general population prevalence, suggesting that choanal narrowing may be a more frequent contributor to airway obstruction in this population than previously recognized. The association between DS and choanal abnormalities is not entirely new. Early case reports by Graham et al. (1981) as well as Itani and Barakat (1986) documented choanal atresia in children with DS, representing foundational clinical observations in the medical literature [7,8].

Despite early recognition of the association between DS and choanal abnormalities in foundational case reports from the 1980s, awareness of this condition appears to remain limited in routine clinical practice. This knowledge gap is reflected in the literature documenting high rates of persistent OSA in children with DS following adenotonsillectomy. Multiple studies demonstrate that 50%-75% of children with DS continue to have residual disease postoperatively [9-11], with cure rates (defined as obstructive apnea-hypopnea index (OAHI) < 2 events/h) ranging from only 16% to 21% compared to approximately 79% in typically developing children [9,12]. Recent research from a large cohort of 75 children with DS found that despite significant improvements in respiratory parameters, approximately 50% retained moderate-to-severe OSA after tonsillectomy [9]. These poor surgical outcomes may partly reflect unrecognized multilevel airway obstruction, including nasal-level pathology such as choanal narrowing, which could contribute to incomplete surgical success in this population.

The persistently high failure rates following adenotonsillectomy in DS underscore the importance of comprehensive airway evaluation when managing persistent OSA symptoms. Current evidence supports the use of drug-induced sleep endoscopy (DISE) to identify multiple sites of upper airway obstruction in children with DS, as these patients demonstrate significantly greater overall obstruction with more frequent tongue base (26% vs 12%) and supraglottic involvement compared to controls [13]. The 2024 American Thoracic Society guidelines for persistent pediatric OSA recommend that DISE with other imaging modalities may be considered when continuous positive airway pressure (CPAP) is not desired or when surgically modifiable sites of obstruction are suspected [14]. Incorporating systematic evaluation of all potential anatomical contributors, including nasal endoscopy to assess the posterior nasal space, aligns with current evidence-based recommendations for multilevel airway assessment and may help identify overlooked pathology that contributes to treatment failures in children with DS and upper airway obstruction.

Limitations of this study include its retrospective design, small sample size, lack of standardized outcome measures, reliance on subjective parental reports, lack of postoperative polysomnography, and inability to control for confounding effects of concurrent procedures. While all patients demonstrated symptom improvement, attribution to choanal narrowing repair cannot be confirmed. The degree of choanal narrowing was estimated intraoperatively using comparative visual assessment with a calibrated 2.7-mm endoscope and a standard suction catheter, performed by an experienced surgeon, which remains inherently subjective and may limit reproducibility. Future studies should include validated outcome tools, such as the OSA-18 questionnaire or polysomnographic data, to provide objective evidence of surgical benefit. Nevertheless, the high prevalence of choanal narrowing in this series suggests a need for prospective, multicenter studies to better define its role in DS airway management.

## Conclusions

Children with DS accounted for 15.5% of patients undergoing choanal narrowing repair at our institution, far exceeding their prevalence in the general population. Notably, 62% were referrals from other pediatric otolaryngologists, highlighting ongoing underrecognition of this pathology. All patients underwent multilevel airway surgery including choanal narrowing repair and had parent-reported symptom improvement. While outcomes cannot be attributed solely to choanal repair due to concurrent procedures, these findings underscore the importance of assessing choanal narrowing during airway evaluation in children with DS. Greater awareness and prospective studies using objective measures are needed to define its specific role in improving outcomes.
